# Exploring the oncogenic roles of T-box transcription factor TBX2 and its potential as a therapeutic target

**DOI:** 10.1042/BST20241069

**Published:** 2025-02-06

**Authors:** Claire Bellis, Mihlali V. Mlaza, Abid Ali, Amaal Abrahams, Sharon Prince

**Affiliations:** Division of Cell Biology, Department of Human Biology, Faculty of Health Sciences, University of Cape Town, Observatory, 7925, Cape Town, South Africa

**Keywords:** TBX2, Oncogene, Targeted therapy, Cancer Biology, T-box transcription factors, Developmental transcription factors

## Abstract

During embryonic development, the T-box transcription factor TBX2 regulates key processes such as cell fate decisions, migration and tissue morphogenesis, and mutations that lead to reduced TBX2 levels result in developmental abnormalities including congenital heart and skeletal defects. TBX2, on the other hand, is overexpressed in a plethora of cancers where it functions as a powerful oncogene contributing to processes ranging from the bypass of senescence and cell death pathways to the promotion of cell proliferation, and epithelial-to-mesenchymal transition to drive invasion and metastasis. Additionally, TBX2 has been implicated in conferring resistance to anti-cancer drugs resulting in poor therapeutic outcomes. To exert its oncogenic functions, TBX2 transcriptionally represses key tumour suppressor genes involved in controlling cell proliferation and epithelial-to-mesenchymal transition such as *p21^Cip1^, p14/p19^ARF^ PTEN, NDRG1, CST6* and *E-cadherin*. This repression has been shown to involve complex mechanisms by which TBX2 co-opts transcription factors and recruits co-repression complexes to the promoters of these tumour suppressor genes. While limited information is available on how TBX2 is regulated in cancers, there is evidence that the levels and oncogenic functions of TBX2 are induced by developmental signalling pathways that are hijacked by cancer cells such as the Wnt/β-catenin and PI3K/AKT pathways. Understanding the complex molecular networks that TBX2 is involved in to exert its oncogenic functions is important because it may reveal potential therapeutic strategies for targeting TBX2 in TBX2-dependent cancers. This minireview discusses TBX2’s involvement in cancer signalling, its regulatory partners, and its impact on cancer progression and resistance to therapy.

## Introduction

**Figure 1:**
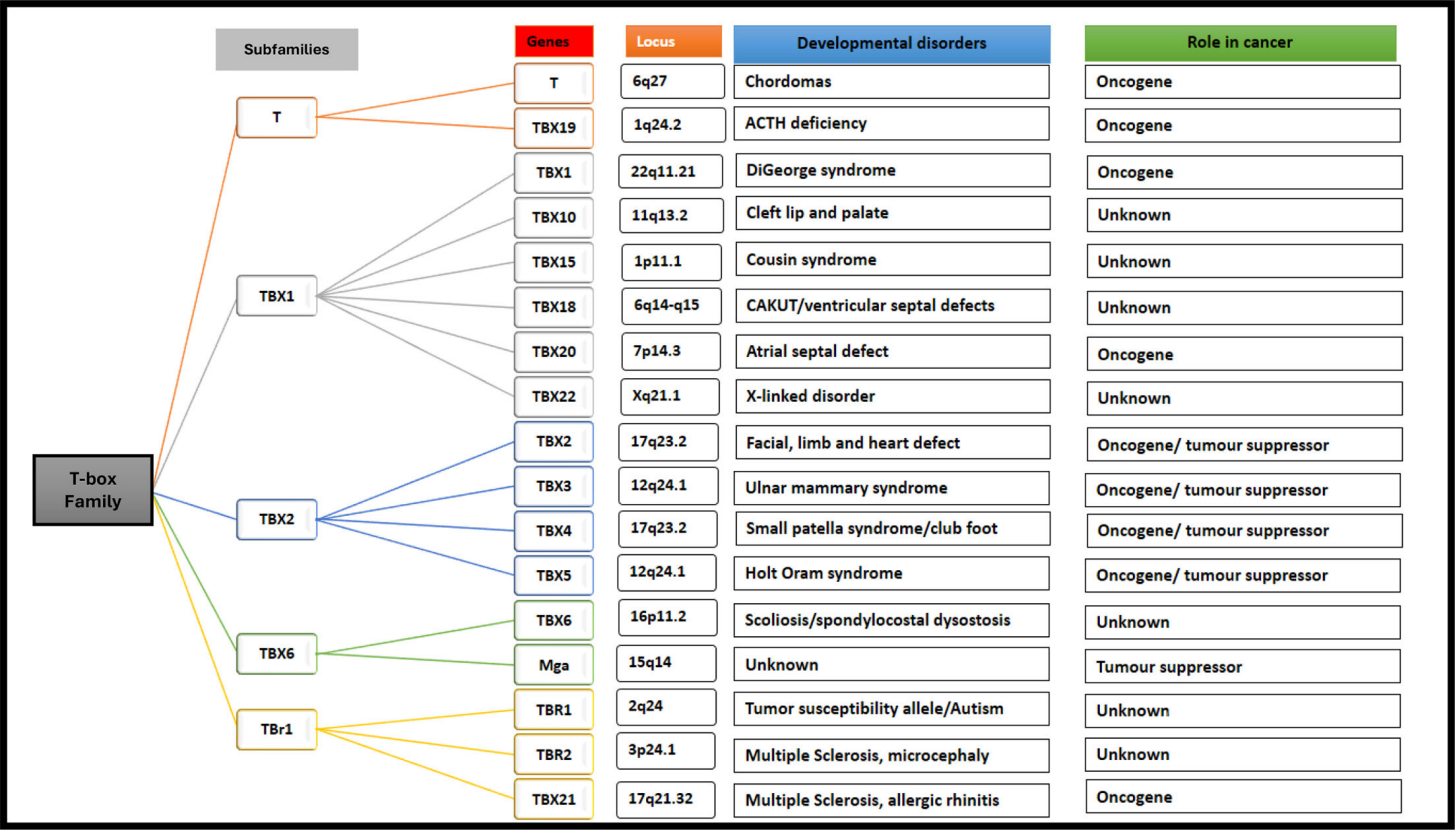
T-box factor gene family and subfamilies. A schematic representation of the various T-box factor subfamilies showing the chromosomal loci of the 17 human T-box factors, developmental disorders associated with mutations in these genes and their role(s) in cancer.

The evolutionarily conserved T-box family of transcription factors plays key roles in embryonic development and their deregulated expression result in developmental defects and diseases, especially cancer (Figure 1). They are divided into five subfamilies (T, TBX1, TBX2, TBX6 and TBr1), and 17 T-box genes have been identified in humans (Figure 1). All T-box factors share a common DNA-binding domain, called the T-box, and they function as either transcriptional activators or repressors with some factors capable of functioning as either one depending on cellular context [1–11].

**Figure 2:**
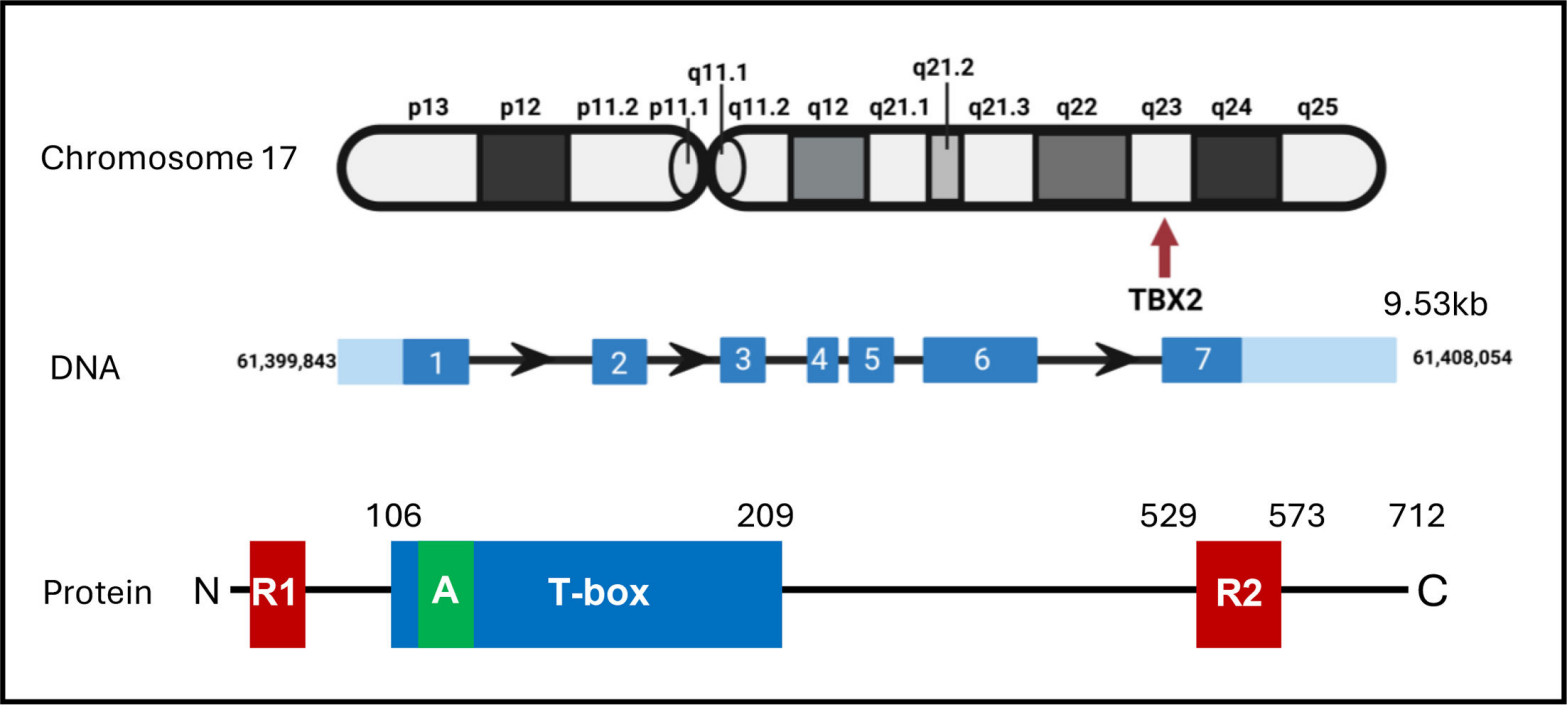
*TBX2* location and gene and protein structure. TBX2 is found at q23 on chromosome 17 in humans. The gene spans 9.53 kb and comprises 7 exons. There is one coding transcript which results in a 712-amino acid protein with two repression domains (R1 and R2; red boxes), a DNA-binding domain, the T-box (blue box) and an activation domain (A; green box) within the T-box.

**Figure 3:**
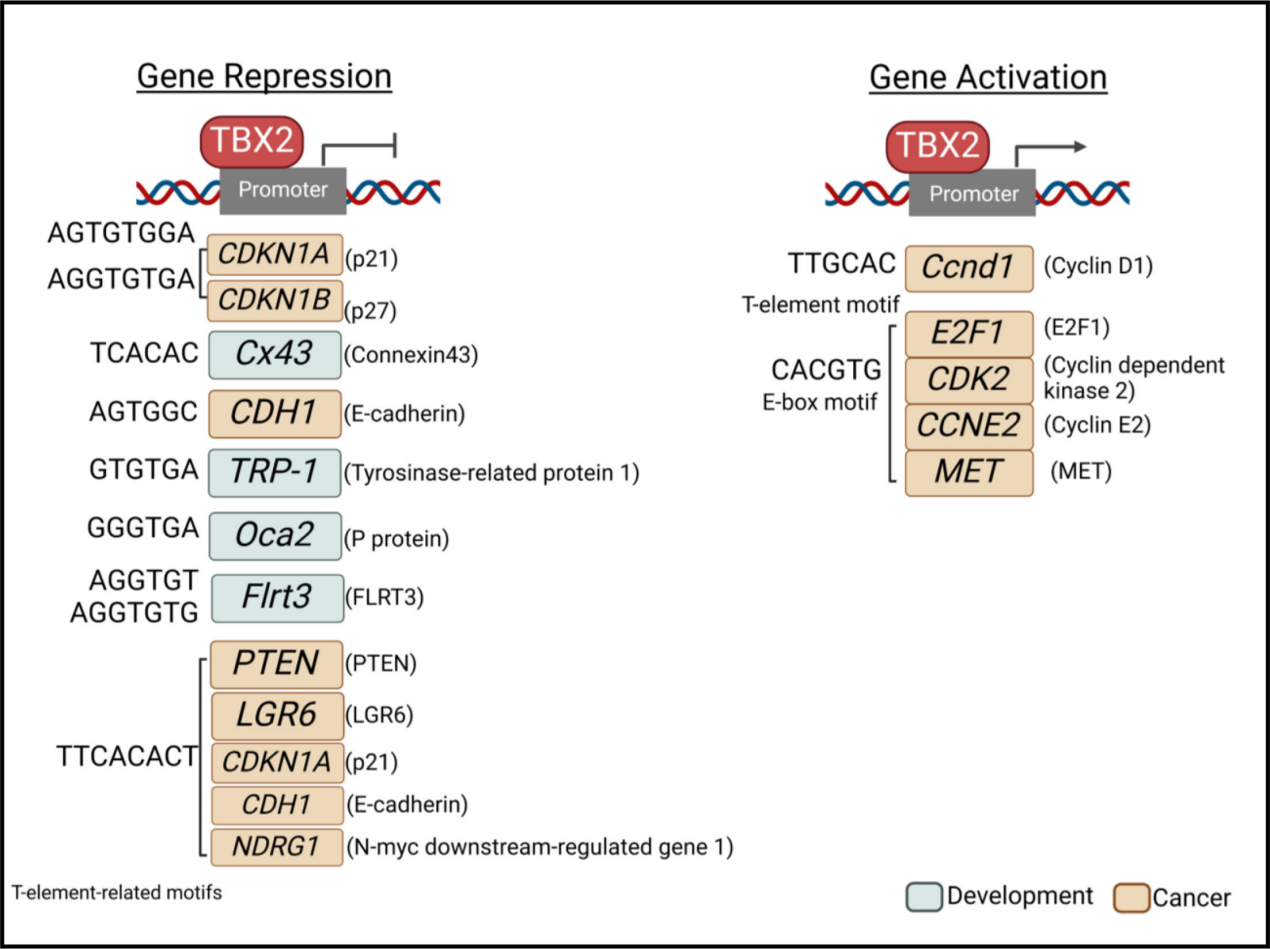
TBX2 target gene regulation. TBX2 represses and activates various genes in development (blue) and cancer (orange). DNA-binding sites are shown for each gene promotor taken from studies where chromatin immunoprecipitation (ChIP) assays were performed.

*TBX2* is a member of the TBX2 subfamily which includes *TBX3*, *TBX4* and *TBX5* (Figure 1). It was the first member of the T-box family to be identified in humans, and the *TBX2* gene was cloned in 1995 [12]. *TBX2* is located on chromosome 17q23, is 9.53 kb in length and has one coding transcript which contains 7 exons (Figure 2). The TBX2 protein is 712 amino acids in length and contains the conserved T-box DNA-binding domain, an activation domain within the T-box, and a repression domain at the N-terminus and another at the C terminus (Figure 2) [3]. TBX2 can transcriptionally activate or repress its target genes through mechanisms involving its ability to bind T-box binding elements (T-elements) or other DNA sequences, such as E-box, EGR1 and Sp1 motifs (Figure 3). Indeed, chromatin immunoprecipitation sequencing (ChIP-seq) carried out in melanoma revealed that, while target genes repressed by TBX2 typically involve T-elements, E-box motifs commonly mediated its activation of its target genes [13]. Similarly, ChIP-seq in breast cancer showed that TBX2 interacted with and co-opted numerous transcription factors, leading to tumour suppressor gene repression through recruitment of the CoREST repression complex [14].

**Figure 4:**
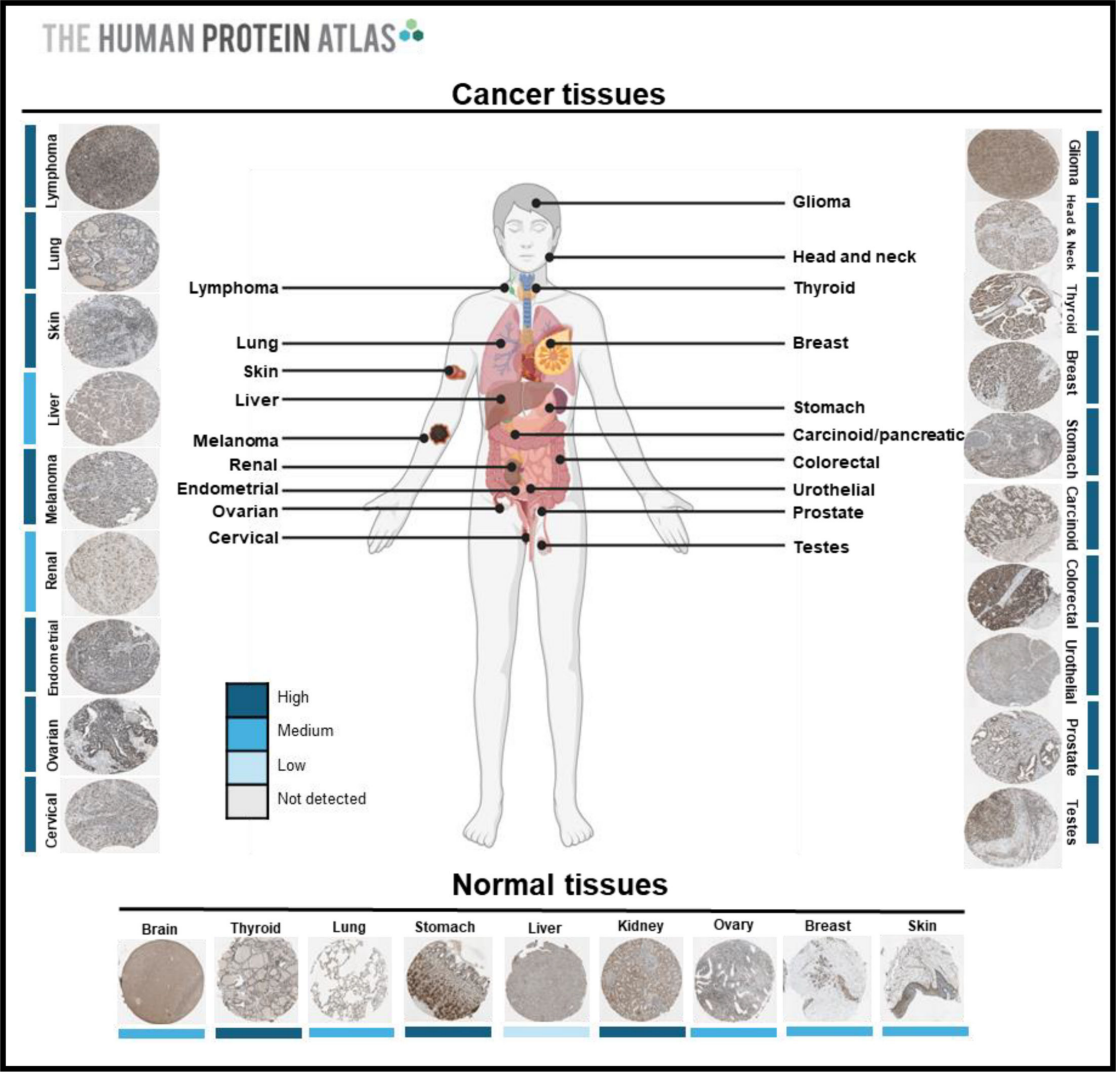
TBX2 is overexpressed in several cancers. The expression of TBX2 in patient samples taken from the Human Protein Atlas is shown relative to normal tissue using the polyclonal rabbit antibody HPA008586 (Sigma-Aldrich). For all tumours except liver and renal carcinomas, TBX2 displayed strong nuclear expression. By contrast, most normal tissues displayed medium intensity staining and for the stomach and kidneys, the high TBX2 expression was mostly cytoplasmic. This figure was created using BioRender.com.

During embryogenesis, TBX2 contributes to the development of several structures, and mutations in *TBX2* result in developmental abnormalities including that of the nervous system, heart, bone, limbs and muscle. While TBX2 has no known function in adult tissue, its postnatal deregulation has been associated with the neurodegenerative disorder, Alzheimer’s disease, and cancer. Indeed, *TBX2* is overexpressed in the cortical tissue of Alzheimer’s patients and plays a pathological role by binding and transcriptionally repressing the metalloprotease, ADAM10, through a process involving histone deacetylase 1 (HDAC1) [15]. Furthermore, *TBX2* is overexpressed in a diverse range of cancers, including melanomas, sarcomas such as malignant peripheral nerve sheath tumours and rhabdomyosarcomas (RMS), and carcinomas of the breast, ovaries, stomach, pancreas, lung, prostate, colorectal, oesophagus, head and neck, and brain Figure 4. The overexpression of *TBX2* frequently correlates with clinical stage and overall poor patient survival and contributes to several cancer hallmarks including proliferation, migration, metastasis, resistance to cell death and conferring drug resistance [16–19]. Not surprisingly, TBX2 has been identified and biologically validated as a promising prognostic marker and drug target in cancers where it is overexpressed.

Several reviews have comprehensively covered the structure of the *TBX2* gene, mRNA and protein, as well as its roles in embryonic development, and hence, this review will focus primarily on the oncogenic roles and regulation of TBX2.

### The role and regulation of TBX2 in proliferation, senescence and cell death pathways

**Figure 5:**
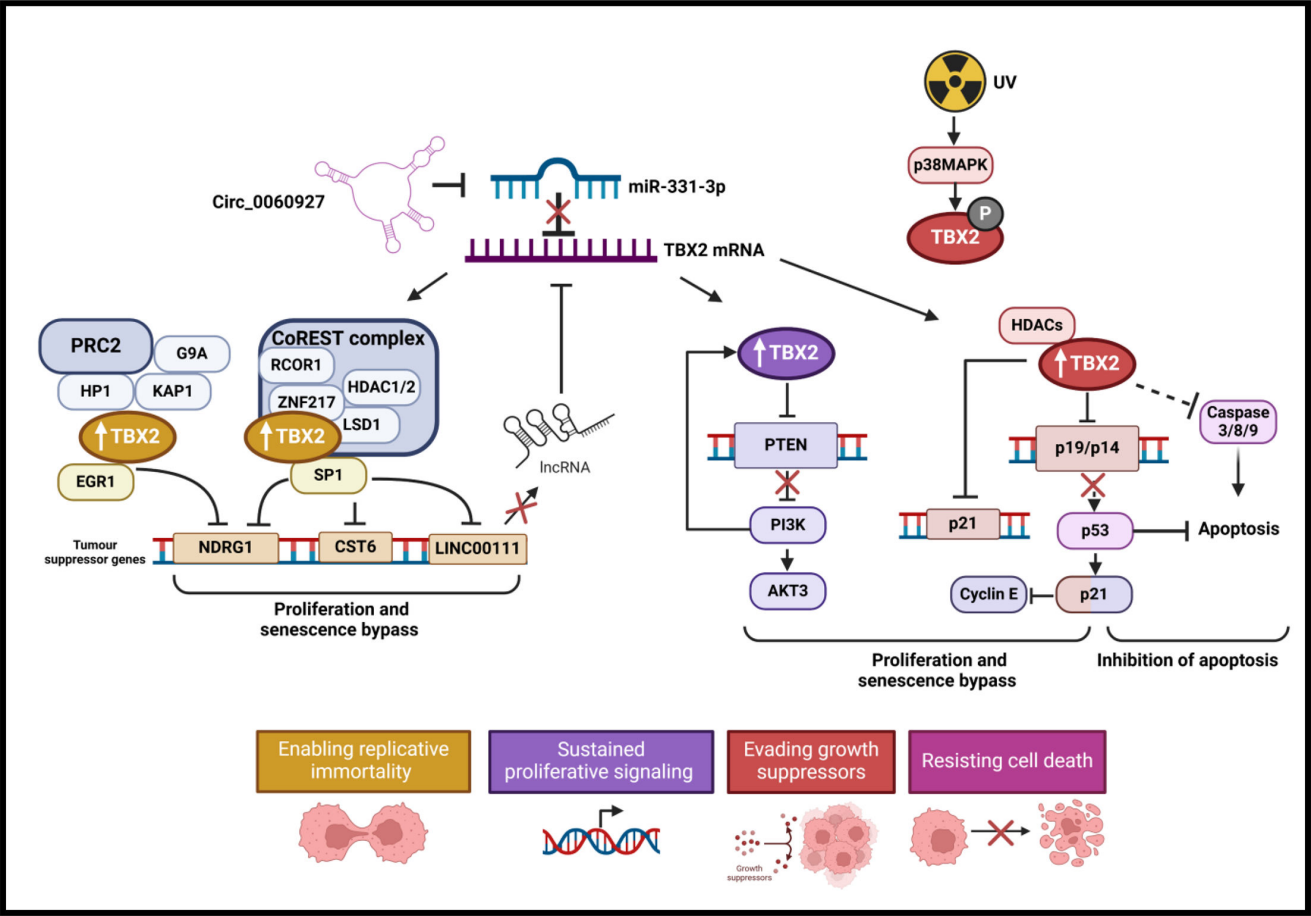
The role and regulation of TBX2 in proliferation, senescence bypass and cell death pathways. TBX2 expression correlates with proliferation and the suppression of apoptosis in cancer cells. TBX2 has been found to be transcriptionally regulated through the Circ_0060927-miR-331–3p axis to promote proliferation and autophagy and to suppress apoptosis. Here sponging of the TBX2 negative regulator miR-331–3p by Circ_0060927 maintains TBX2 levels in the cell. UV has also been shown to promote phosphorylation of TBX2 by p38 MAPK. Mechanistically, TBX2 co-operates with repressive complexes CoREST and PRC2 and co-factors SP1 and EGR1 to suppress critical growth suppressor genes *NGRD1, CST6 and LINC00111* enabling replicative immortality and the evasion of growth suppressors. LINC00111 has also been proposed as a negative regulator of TBX2. Additionally, TBX2 directly represses *PTEN* to activate PI3K/AKT signalling promoting sustained proliferative signalling which further promotes TBX2 expression through positive feedback. TBX2 also directly suppresses the cell cycle genes *p21^Cip1^* and *p19/p14^ARF^* to promote cell proliferation. The activation of p53 downstream of p19/p14 facilitates the inhibition of apoptosis and TBX2 levels inversely correlate with caspase 3/8/9 levels. This figure was created using BioRender.com.

Uncontrolled proliferation, a key hallmark of cancer, occurs when cells acquire the ability to bypass key cell cycle checkpoints that ordinarily serve as barriers to cancer [20]. This includes the ability to evade growth suppressors, which can occur through the down-regulation of tumour suppressors or bypass of senescence and resisting cell death processes such as apoptosis [21]. TBX2 has been shown to function as a key oncogenic factor through its ability to contribute to these processes by repressing tumour suppressor genes Figure 5. Indeed, Jacobs and colleagues showed that *TBX2* overexpression promoted the immortalisation of *Bmi-null*- primary mouse embryo fibroblasts through the repression of *Cdkn2a (p19^ARF^),* delaying the onset of senescence. Similarly in melanoma, *Tbx2* overexpression directly repressed *p21^CIP1^* which inhibited the onset of replicative senescence and promoted cell cycle progression [22,23]. Mechanistically, this was shown to involve Tbx2’s ability to recruit HDAC1 to the *CDKN1A* initiator [23]. A similar mechanism was reported in RMS where TBX2 drives cancer progression by inhibiting myogenesis in favour of promoting proliferation [24]. TBX2 achieved this by recruiting HDAC1 to the promoters of the myogenic regulatory factors, *MyoD* and *myogenin*, and the tumour suppressors, *p21^Cip1^, p14^ARF^* and *PTEN* [24,25]. In nasopharyngeal cancer cells, TBX2 levels inversely correlated with the tumour suppressors PTEN, p21^Cip1^, p27^Kip1^ and E-cadherin, and knocking down TBX2 led to their up-regulation and a significant inhibition of proliferation and invasion [26].

Several lines of evidence suggest that the mechanism by which TBX2 represses tumour suppressor genes to promote uncontrolled proliferation and senescence bypass involves co-opting other transcription factors Figure 5. For example, TBX2 interacts with and requires early growth response 1 (EGR1) to inhibit the tumour suppressor genes *N-myc downstream-regulated gene 1* (*NDRG1*) and *cysteine protease inhibitor cystatin 6* (*CST6*) to promote breast cancer cell proliferation [27,28]. A subsequent study showed that the mechanism involved the recruitment of a large repression complex in which TBX2 interacts with heterochromatin protein 1 (HP1), which in turn leads to the recruitment of KRAB domain-associated protein 1 (KAP1) and components of polycomb repressive complex 2 (PRC2) to the promoters of TBX2/EGR1 co-regulated genes [29]. Interestingly, in RMS, PRC2 represses *TBX3* which prevents it from transcriptionally repressing *TBX2*. This results in the de-repression of *TBX2* and, consequently, the repression of the TBX2 target tumour suppressor genes *p21^Cip1^/p14^ARF^/PTEN* and the promotion of proliferation and bypass of apoptosis [30]. Recently, TBX2 was also shown to repress *NDRG1* and *CST6* in breast cancer by recruiting a CoREST repression complex (LSD1, ZNF217 and HDAC1) to their promoters [30]. It is worth noting that the regulation of *NDRG1* by the TBX2-CoREST complex was shown to be dependent on Sp1 recruitment. Depleting TBX2, Sp1 or CoREST members resulted in the de-repression of *NDRG1* which was accompanied by reduced breast cancer cell viability and colony-forming ability. Importantly, pharmacological inhibition of LSD1 disrupted the CoREST function and reduced tumour growth *in vivo*. Together, these findings highlighted the essential role that the TBX2-CoREST-Sp1 axis plays in maintaining breast cancer survival and revealed its therapeutic potential. The same study showed that the TBX2-CoREST complex transcriptionally represses the long non-coding RNA LINC00111 through a region containing a CREM-binding motif, leading to breast cancer cell growth and survival. Interestingly, siRNA knockdown of LINC00111 resulted in TBX2 up-regulation suggesting the existence of reciprocal repression of TBX2 by LINC00111 Figure 5.

In melanoma, TBX2 was shown to promote proliferation and senescence bypass downstream of the PI3K signalling pathway where it activates the expression of the anti-senescence cell cycle regulator *E2F1* and represses *PTEN*, a negative regulator of the AKT/PI3K pathway [13]. TBX2 was shown to activate *E2F1* expression by recruiting key components of the BCOR/PRC1.1 complex to the *E2F1* locus [13]. In RMS, TBX2 also represses *PTEN*, suggesting a self-regulatory loop where TBX2 helps maintain the anti-senescence signal [24]. In breast cancer cells, stress signals initiated by ultraviolet-C irradiation promoted the phosphorylation of TBX2 by p38 MAP kinase, resulting in an increase in TBX2 protein levels, nuclear localisation and its ability to repress *p21^Cip1^* [31]. A study conducted by Ismail and Bateman also showed that TBX2 might be post-transcriptionally modulated by the AKT pathway in adrenal carcinoma cell lines with no observable amplifications of the 17q23 locus [32]. The overexpression of *TBX2* also correlates with colorectal cancer (CRC) disease progression and CRC cell proliferation [33–35]. Yin et al. showed that this overexpression results from the circular RNA circ_0060927 sponging miR-331–3p, which typically represses TBX2 [33]. Functionally, this promoted proliferation and autophagy and inhibited apoptosis and necrosis [33].

Together, the above studies suggest that TBX2 bypasses senescence, promotes proliferation and evades cell death programmes through complex mechanisms involving its ability to hijack other transcription factors and recruit repression complexes to the promoters of tumour suppressor genes Figure 5. More in-depth studies are required to confirm if these mechanisms are relevant to all TBX2-dependent cancers or if there are other novel molecular mechanisms involved.

### The role and regulation of TBX2 in anoikis, epithelial–mesenchymal transition, invasion and metastasis

**Figure 6:**
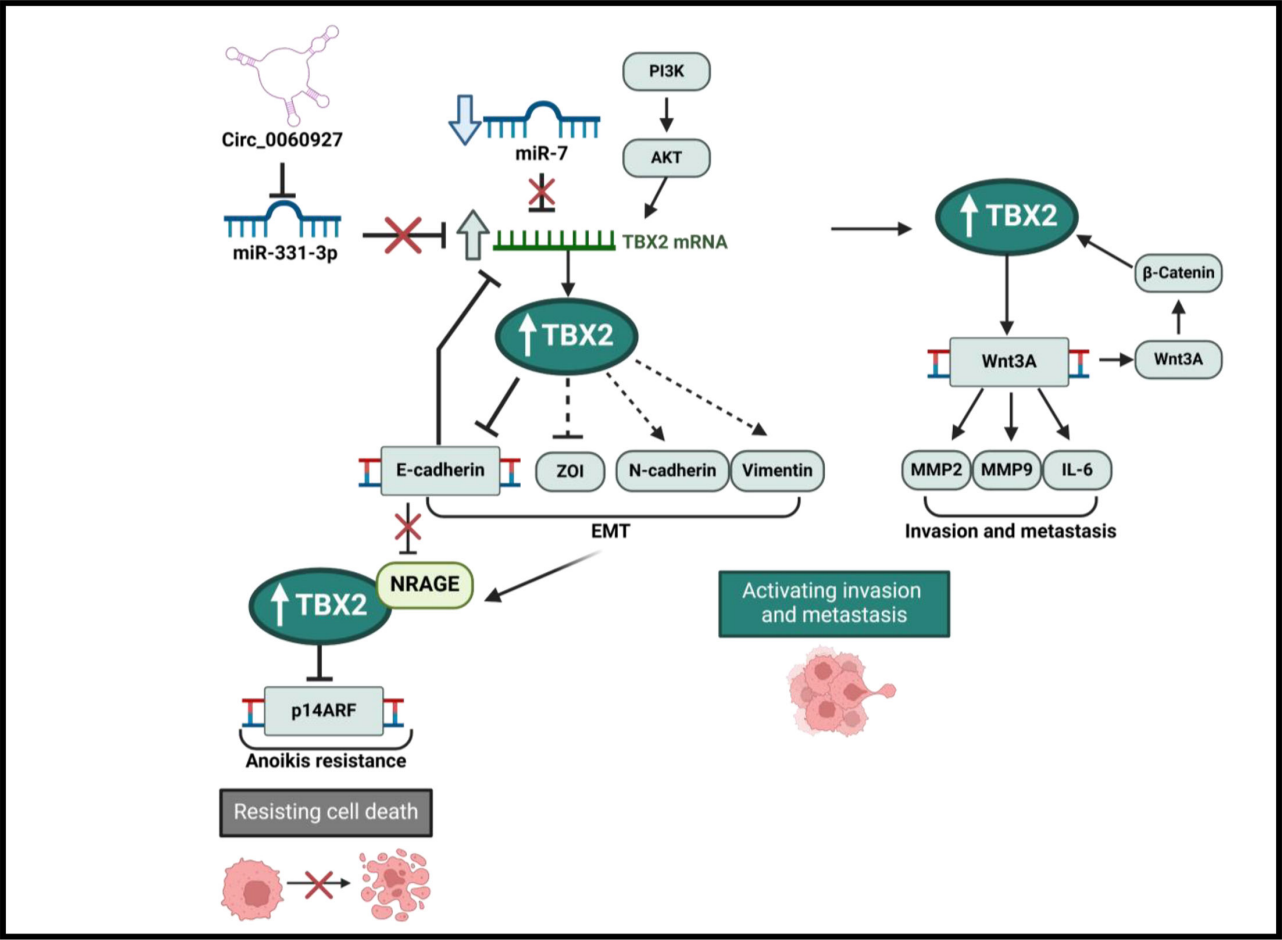
The role and regulation of TBX2 in anoikis, epithelial–mesenchymal transition, invasion and metastasis. TBX2 has been shown to directly repress *E-cadherin* in several tumour subtypes to promote EMT. E-cadherin reciprocally inhibits *TBX2* mRNA levels in a negative feedback loop. In the pre-EMT state, E-cadherin represses NRAGE to promote anoikis sensitivity. Conversely, following EMT, loss of E-cadherin releases NRAGE allowing it to interact with TBX2. The TBX2-NRAGE complex represses p14ARF and promotes resistance to anoikis, resulting in cancer cell survival. TBX2 expression has also been found to inversely correlate with the EMT marker ZOI, and TBX2 expression promotes expression of the mesenchymal markers N-cadherin and vimentin. In some tumours, microRNA repressors of TBX2 are consistently suppressed which leads to the up-regulation of TBX2. In some tumours, TBX2 has been shown to directly activate *WNT3A* facilitating metastasis through the up-regulation of WNT3A, targeting MMP2, MMP9 and IL-6. At the same time, Wnt activates β-catenin, creating a feedback loop that up-regulates TBX2 levels. This figure was created using BioRender.com.

Epithelial–mesenchymal transition (EMT), invasion and migration are key processes that facilitate cancer metastasis and progression, and TBX2 has been found to promote these processes in several cancers. Indeed, TBX2 expression correlates with poor prognosis in glioblastoma (GBM) and CRC; invasion and metastasis in lung, breast, pancreatic, prostate, nasopharyngeal, gastric and CRC; tumour recurrence in gastric cancer; and lymph node metastasis in cervical cancer and endometrial adenocarcinoma [26,33–46]. The mechanisms by which TBX2 facilitates EMT, invasion and migration are outlined below and summarised in Figure 6.

During oncogenic EMT, cells suppress anoikis, a type of programmed cell death, to lose attachment to the extracellular matrix and neighbouring cells which results in invasion and metastasis. Kumar and co-workers demonstrated that TBX2 in association with the neurotrophin receptor-interacting melanoma antigen (NRAGE) repressed *p14^ARF^* to confer resistance to anoikis during the EMT transition [47]. Morphologically, EMT is characterised by epithelial cells acquiring mesenchymal features which are necessary for the invasive and metastatic capacity of tumours. At a molecular level, EMT is associated with the loss of adherent junctions and the down-regulation of cytokeratins and E-cadherin, along with an increase in mesenchymal markers, such as N-cadherin and β-catenin [48]. Wang and co-workers first demonstrated that TBX2 is a strong inducer of EMT in breast cancer [39]. They showed that the ectopic expression of TBX2 in normal mammary epithelial cells resulted in the loss of epithelial adhesion and polarity markers such as E-cadherin, β -catenin and tight junction protein-1 (ZO1), and induced a significant increase in N-cadherin and Vimentin. Conversely, inhibiting TBX2 in breast cancer, prostate cancer, oesophageal squamous cell carcinoma and lung adenocarcinoma abrogated the expression of mesenchymal markers with reciprocal restoration of the epithelial state [38,39,42,49]. In CRC, TBX2 overexpression also resulted in the activation of EMT markers, increased invasion and promoted tumourigenesis *in vivo* [34,35]. Interestingly, in osteosarcoma, E-cadherin overexpression resulted in a reduction in *TBX2* mRNA levels, suggesting the possible involvement of a negative feedback loop during EMT [50]. Overall, the loss of TBX2 was found to inhibit breast, prostate and oesophageal cancer cell invasion and migration [42,49]. In this regard, it is worth noting that a recent study demonstrated a novel mechanism by which TBX2 can be targeted to inhibit EMT in GBM. MicroRNA-7 (miR-7) is commonly down-regulated in GBM, and the authors noted that the levels of TBX2 and miR-7 were negatively correlated, and that miR-7 targeted TBX2 for degradation by binding its 3′-UTR. This led to an increase in E-cadherin and a decrease in Vimentin which resulted in an inhibition of EMT and invasion [51]. As mentioned earlier, Yin et al. also showed that TBX2 is overexpressed in CRC due to circular RNA circ_0060927 sponging miR-331–3p. Relevant to this section, their study showed that this resulted in the ability of TBX2 to promote cCRC cell, invasion and migration [33].

Some studies have hinted at possible molecular pathways underpinning the pro-invasive and pro-migratory capacity of TBX2 [36,43,51]. For example, the PI3K/AKT and Wnt/β-catenin signalling pathways play key roles in the EMT process, and both these pathways were shown to converge on TBX2 in some cancers. Indeed, in adrenocortical carcinoma, the PI3K/AKT pathway led to an increase in TBX2 mRNA and protein levels, which consequently promoted anchorage-independent growth [32]. Furthermore, TBX2 is overexpressed in pancreatic cancer tissues and cell lines, and its expression increased with β-catenin accumulation [52]. Interestingly, in prostate and CRC, TBX2 transcriptionally activates *WNT3A* which in turn activates matrix metalloproteinases (MMPs), particularly MMP2 and MMP9, as well as IL-6 to induce invasion [35,43]. It is worth noting that in gastric cancer, the overexpression of TBX2 also led to the up-regulation of MMP-2 and -9 and, while undefined, this may be occurring through TBX2 activation of *WNT3A* [36]. Together, these studies suggest that, at the very least in some cancers, there is a positive feedback loop between the Wnt/β-catenin pathway and TBX2 which drives invasion and migration.

Collectively, the above findings reveal a critical role for TBX2 in oncogenic EMT, invasion and metastasis downstream of the Wnt/ β catenin and PI3K/AKT pathways.

### The role and regulation of TBX2 in drug resistance

**Figure 7:**
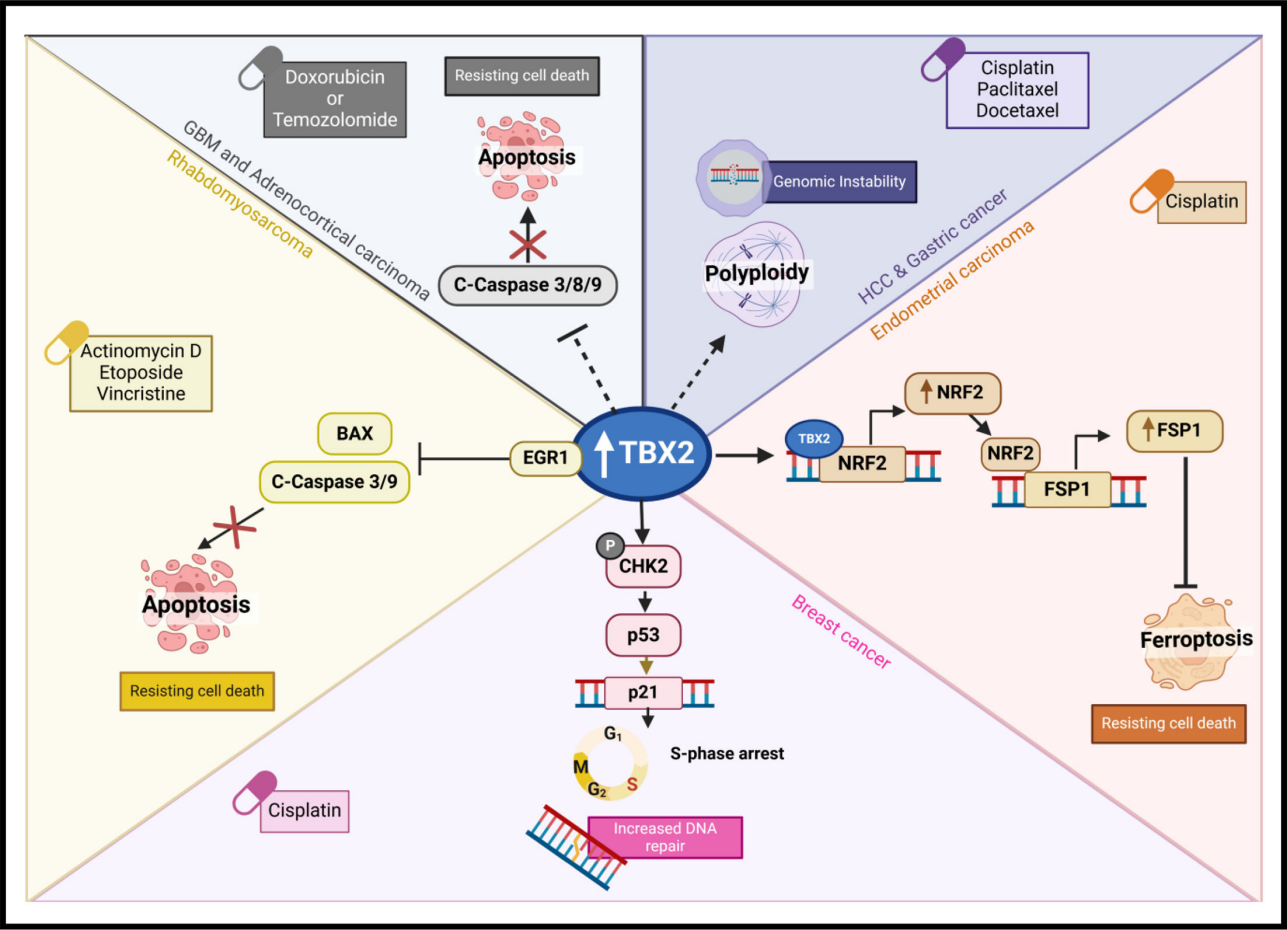
The role and regulation of TBX2 in drug resistance. TBX2 overexpression has been shown to facilitate drug resistance in several cancers. In response to actinomycin D, etoposide and vincristine treatment, TBX2 interacts with EGR1, inhibiting its ability to promote apoptotic gene expression (BAX and c-caspase 3/9). This prevents apoptosis and facilitates drug resistance. Similarly, in response to doxorubicin treatment, TBX2 expression negatively correlated with cleavage of caspases 3, 8 and 9, resulting in the suppression of apoptosis. TBX2 also suppresses temozolomide-induced apoptosis in glioblastoma (GBM). In hepatocellular carcinoma (HC) and gastric cancer, TBX2 promotes resistance to a cisplatin, paclitaxel and docetaxel regimen by inducing polyploidy. Finally, TBX2 facilitates cisplatin resistance by promoting DNA repair via the pCHK2-p52-p21^Cip1^ axis and by suppressing ferroptosis via the NRF2-FSP1 axis. This figure was created using BioRender.com.

Intrinsic or acquired tumour drug resistance continues to pose a major burden in the clinical management of cancer patients [53]. The identification of predictive biomarkers and molecular drivers that contribute to drug resistance is therefore essential. This will aid with administering appropriate therapies to patients and with the development of effective anti-cancer therapies [18]. Accumulating evidence has implicated TBX2 in tumour drug resistance (Figure 7. For example, in astrocytoma and GBM TBX2, overexpression correlated with resistance to temozolomide, the gold standard for GBM treatment [37]. Similarly, TBX2 expression was significantly up-regulated in platinum-resistant ovarian cancer patients, cisplatin-resistant endometrial cancer patients and in poor responders to postoperative adjuvant chemotherapy in stage II/III gastric cancer [18,46,54]. Importantly, the knockdown of TBX2 promoted sensitivity to cisplatin in breast cancer and melanoma, to temozolomide in GBM, to carboplatin in ovarian serous carcinoma and to androgen deprivation therapy in advanced prostate cancer [17–19,37].

Mechanistically, TBX2 was found to promote resistance to first-line chemotherapeutics by promoting polyploidy, inhibiting apoptosis and ferroptosis, and promoting DNA damage repair as summarised in **Figure 7** [16,17,32,55]. Indeed, the overexpression of TBX2 was reported to confer cisplatin resistance in transformed lung fibroblasts by inducing genomic instability and polyploidy [16]. Consistent with this, TBX2 up-regulation is associated with drug resistance and the induction of polyploidy in mouse hepatocellular carcinoma and human gastric cancer cells treated with a combination of cisplatin and paclitaxel or cisplatin, paclitaxel and docetaxel [55]. Moreover, in adrenocortical carcinoma cells, TBX2 expression promoted resistance to the first-line treatment, doxorubicin, by modulating caspase expression and thus inhibiting apoptosis [32]. In RMS, TBX2 was also reported to promote resistance to actinomycin D, etoposide and vincristine through its ability to inhibit the expression of EGR1-dependent cell cycle regulators and pro-apoptotic target genes [56]. TBX2 was also shown to confer cisplatin resistance in breast cancer cells by activating CHK2 to ensure the phosphorylation and stability of p53 which enhances its transcriptional activation of *p21^Cip1^* [17]. This resulted in the induction of an S-phase cell cycle arrest which allowed for DNA damage repair and cancer cell survival. The same study showed that reducing TBX2 levels in cisplatin-resistant breast cancer cells led to mitotic catastrophe and drug sensitivity. In addition, TBX2 was shown to promote cisplatin resistance in endometrial carcinoma by inhibiting ferroptosis, a form of regulated cell death, and accelerating tumour growth in cisplatin-treated mice [54,57]. This was achieved by TBX2 transcriptionally up-regulating the antioxidant transcription factor *nuclear factor erythroid 2-related factor 2* (*NRF2*) and enhancing expression of ferroptosis suppressor protein 1 (FSP1), a NRF2 target gene. Importantly, knockdown of TBX2 restored sensitivity to cisplatin-induced ferroptosis.

Taken together, these studies support the development of TBX2 as a predictive biomarker of treatment responses, particularly in the case of platinum-based compounds, and suggest that combining a TBX2-targeted approach with chemotherapies like cisplatin has the potential to enhance the overall success of current cancer treatments.

### Conclusions and future implications

TBX2 plays diverse and critical roles in cancer biology from promoting tumourigenesis by driving cell proliferation, senescence bypass and apoptosis inhibition, to driving EMT, invasion and migration. Additionally, TBX2 has emerged as a significant contributor to drug resistance, particularly in response to DNA-damaging agents. This positions TBX2 as an attractive target for therapeutic intervention in a wide range of carcinomas, sarcomas and melanoma. However, to the best of our knowledge, there are only two reports that have identified anti-cancer agents that function specifically through their ability to inhibit TBX2. The one used a reverse affinity approach to screen libraries of natural products from marine bacterial species that bind to and inhibit the oncogenic activity of TBX2. In this screen, the aureolic acid chromomycin A5 (CA5) was identified and its interaction with TBX2 was confirmed using microscale thermophoresis. Importantly, CA5 was found to display potent cytotoxicity against TBX2-driven melanoma, breast and RMS cells, and in melanoma, this was shown to involve its ability to target TBX2 [58]. To identify rapid and cost-effective drugs that target TBX2, the other report described a combined high-throughput target-based approach with a drug repurposing screen with the aim of identifying commercially available drugs that specifically target TBX2 and/or its homolog, TBX3 for the treatment of cancer [59]. Niclosamide, piroctone olamine and pyrvinium pamoate were identified as ‘hit’ drugs that exhibit cytotoxicity in melanoma and RMS through their ability to target TBX2 and TBX3. This is important because TBX2 and TBX3 can transcriptionally repress one another in certain contexts, and they have been shown to have distinct oncogenic roles in cancers where they are both expressed [60]. For example, in melanoma and breast cancer, TBX2 functions as a pro-proliferative and anti-senescence factor, and TBX3 promotes migration and invasion [60]. To effectively treat these TBX2/TBX3-dependent cancers, it would, therefore, be important to block both TBX2 and TBX3 because inhibiting only one of them would result in the up-regulation of the other. As repurposed drugs, niclosamide, piroctone olamine and pyrvinium pamoate could be rapidly progressed to the clinic as anti-cancer drugs, but first their safety and efficacy should be urgently investigated in pre-clinical models.

Future research should focus on identifying other compounds that directly interact with and inhibit the oncogenic functions of TBX2. This would be facilitated by elucidating the three-dimensional structure of TBX2 because it will provide information of drugs that can bind to TBX2 and reveal ways of manipulating these drugs to better inhibit its oncogenic functions. Furthermore, to identify versatile ways of targeting TBX2, it will be important to expand on our knowledge regarding the post-translational modification profile of TBX2 and to further elucidate epigenetic and oncogenic signalling pathways that up-regulate TBX2 levels, as well as protein co-factors and target genes that mediate its oncogenic functions. In this regard, it is worth noting that there are inhibitors to known TBX2 co-factors (e.g. HDAC1/2 and Sp1) and upstream regulators (e.g. PI3K/AKT and Wnt/β-catenin) that are already FDA-approved or currently undergoing clinical evaluation for their therapeutic potential in cancer treatment. Indeed, there are several pharmacological inhibitors to components of the CoREST transcriptional corepressor complex that TBX2 interacts with, such as histone deacetylases HDAC1/2 (e.g. the pan-HDAC inhibitors vorinostat, romidespin, panobinostat and belinostat for the treatment of haematological cancers) and the histone demethylase LSD1 (e.g. RG6016 and TCP/ATRA) [61–63]. More recently, corin, a hybrid agent derived from the HDAC inhibitor entinostat and a tranylcypromine analog LSD inhibitor, has also been identified as a dual inhibitor of both HDAC1/2 and LSD1 with efficacy in several cancers including melanoma and breast cancer [64,65]. Finally, Sp1 recruits the TBX2-CoREST complex to the promotor of pro-proliferative genes, and the well-known chemotherapeutic drugs doxorubicin and mithramycin function in part by preventing the binding of Sp1 to DNA [66–71]. Additionally, natural compounds such as tolfenamic acid and curcumin have been reported to reduce Sp1 levels in *in vitro* and *in vivo* cancer models [72,73]. Pharmacological inhibitors of CoREST components, particularly HDAC1/2 and LSD1, and Sp1, offer promising avenues for suppressing TBX2-dependent tumorigenesis.

Upstream regulators of TBX2 include PI3K/AKT and Wnt/β-catenin, and inhibitors to these pathways have been identified that demonstrate efficacy in several cancers. These include the PI3K/AKT inhibitors copanlisib and alpelisib for the treatment follicular lymphoma and metastatic breast cancer, respectively [74–76]. Interestingly, niclosamide, piroctone olamine and pyrvinium pamoate inhibit the Wnt/β-catenin pathway and were recently shown to inhibit TBX2 and TBX3 levels and to exert anti-cancer activity in TBX2/3-driven melanoma and rhabdomyosarcoma [59,77–83]. There are, therefore, examples of drugs that can exert anti-cancer activity, in part, through inhibiting multiple components of the TBX2 oncogenic pathway. Another strategy to inhibit TBX2 could be through microRNA replacement therapy, for example through restoring the tumour suppressors miR-7 and miR-331–3p, known negative regulators of TBX2. A better understanding of how TBX2 confers resistance to a range of cancer treatments could also inform the feasibility of combining TBX2 inhibitors with other therapies such as DNA-damaging agents.

Finally, the development of approaches, such as proteolysis targeting chimeras, clustered regularly interspaced short palindromic repeats gene editing and artificial intelligence, as well as advances in structural biology, can now be utilised to effectively target transcription factors like TBX2 which have historically been considered undruggable or hard to target.

**Perspectives**

- TBX2 is a developmental transcription factor that is overexpressed in numerous cancers where it functions as a powerful oncogene by driving tumour progression, invasion and metastasis, and conferring tumour drug resistance. It accomplishes these functions by repressing key tumour suppressor genes involved in cell cycle control and epithelial adhesion.
- Depleting TBX2 in cancers addicted to it inhibits key hallmarks of cancer. This positions TBX2 as an attractive target for therapeutic intervention in a wide range of carcinomas, sarcomas and melanoma.
- Future research should identify versatile ways of targeting TBX2, for example, by determining its three-dimensional structure and post-translational modification profile, as well as elucidating the epigenetic and signalling pathways that up-regulate TBX2 levels and the protein co-factors and target genes that mediate its oncogenic functions.

## Open Access

This article has been published open access under our Subscribe to Open programme, made possible through the support of our subscribing institutions, learn more here: https://portlandpress.com/pages/open_access_options_and_prices#conditional

## Abbreviations

CA5: chromomycin A5
CHK2: Checkpoint kinase 2
CRC: colorectal cancer
CRISPR: Clustered regularly interspaced short palindromic repeats
CST6: cysteine protease inhibitor cystatin 6
ChIP-seq: chromatin immunoprecipitation sequencing
ECM: Extracellular matrix
EGR1: early growth response 1
EMT: epithelial–mesenchymal transition
FSP1: ferroptosis suppressor protein 1
GBM: glioblastoma
HDAC1: histone deacetylase 1
HP1: heterochromatin protein 1
KAP1: KRAB domain-associated protein 1
MAP: mitogen-activated protein kinase pathway
MMPs: matrix metalloproteinases
NDRG1: N-myc downstream-regulated gene 1
NRAGE: neurotrophin receptor-interacting melanoma antigen
NRF2: Nuclear factor erythroid 2-related factor 2
PI3K: phosphatidylinositol 3-kinase
PRC2: polycomb repressive complex 2
PROTACs: Proteolysis Targeting Chimeras
PTEN: Phosphatase and tensin homolog
RMS: rhabdomyosarcomas
SP1: Specificity protein 1
TBX2: T-box transcription factor 2
T-elements: T-box binding elements
UTR: untranslated region
miR-7: MicroRNA-7
miR-331-3p: MicroRNA-331-3p
